# The emerging role of histone deacetylase 1 in allergic diseases

**DOI:** 10.3389/fimmu.2022.1027403

**Published:** 2022-10-11

**Authors:** Yongfang Wang, Huiying Wang

**Affiliations:** Department of Allergy, Second Affiliated Hospital, Zhejiang University School of Medicine, Hangzhou, China

**Keywords:** epigenetic modifications, allergic diseases, HDAC1, Th2 cytokines, IL-10, Trek-1

## Abstract

Histone deacetylase 1 (HDAC1) is a unique member of the classes I HDACs and helps to regulate acute and chronic adaptation to environmental stimuli such as allergen, stress. Allergic diseases are complex diseases resulting from the effect of multiple genetic and interacting foreign substances. Epigenetics play an important role in both pathological and immunomodulatory conditions of allergic diseases. To be consistent with this role, recent evidence strongly suggests that histone deacetylase 1 (HDAC1) plays a critical role in allergic response. HDAC1 expression is stimulated by allergen and attributes to increase T helper 2 (Th2) cytokine levels, decrease Th1/Th17 cells and anti-inflammatory cytokine Interleukin-10 (IL-10), and TWIK-related potassium channel-1 (Trek-1) expression. This review focuses on the contribution of HDAC1 and the regulatory role in characterizing allergic endotypes with common molecular pathways and understanding allergic multimorbidity relationships, as well as addressing their potential as therapeutic targets for these conditions.

## Introduction

Epigenetics includes the heritable alterations in gene expression without any changes in a deoxyribonucleic acid (DNA) sequence, which is crucial in the pathophysiology of many diseases (1, 2). Multiple enzymes have been extensively studied that induce epigenetic changes, such as DNA methylation and histone acetylation of DNA regions. Histone deacetylases (HDACs) are the enzymes that catalyze lysine deacetylation of both histone and non-histone proteins. HDACs increase the positive charge on histones after removing acetyl groups from lysine residues, thus increasing the affinity of positively charged histones for negatively charged DNA (3). HDACs lead to the condensation of the chromatin and then reduces the accessibility of transcriptase, and finally leads to an overall suppression of gene transcription (4). HDAC family has four subclasses including I, II, III and IV. Classes I, II, and IV HDACs utilize a zinc-dependent mechanism and belong to the Zn2+ superfamily, while class III HDACs require nicotinamide adenine dinucleotide(NAD)+ for catalytic activity.

Histone deacetylase 1 (HDAC1) is a unique member of the classes I HDACs that has been shown to be involved in gene transcription, transcriptional regulation, cell cycle progression and developmental events by controlling both enzyme activity and epigenetics of key proteins (5). HDAC1 is the most abundant member of the class I HDACs in pulmonary endothelial cells (6), regulating the enzymatic activity and epigenetics of key proteins to adapt to external stimuli. It can efficiently decrotonylate this relatively less abundant histone modification (7). Moreover, HDAC1 is the key regulators of T cell subset differentiation and T cell-mediated immune diseases (8) that helps to regulate acute and chronic adaptation to environmental stimuli such as allergen, stress (9). Allergic diseases represent a collection of disorders such as allergic rhinitis, asthma, that mostly characterized by a type 2 immune response involving Th2 cells, eosinophils and mast cells, and M2 macrophages. T cell specific loss of HDAC1 leads to an increase in Th2 type allergic airway inflammation, such as enhanced secretion of Th2 type cytokines, eosinophil recruitment to the lung (10).For example, HDAC1 is highly expressed and the most abundant member of the class I HDACs in allergic rhinitis and severe asthma (3, 11, 12). Studies show that HDAC1 is localized within most airway cells and infiltrating inflammatory cells of asthmatic lung tissues (13). HDAC1 is significantly upregulated in the murine AR model while H3 acetylation is decreased at lysine 9 (H3AcK9) (14). The HDAC1 inhibitor sodium butyrate exhibits a preventive effect by decreasing HDAC1 expression and increasing H3 acetylation at lysine 9. Herein, we made a thorough review of recent studies and summarized the emerging functions of HDAC1 by regulating histone modifications and gene transcription in allergic disease.

## Allergic diseases

Generalized allergic diseases include allergic rhinitis, asthma, Immunoglobulin E(IgE)-mediated food allergy, eosinophilic esophagitis, drug allergy, atopic dermatitis, and urticaria/angioedema. These different allergic diseases share several overlapping inflammatory pathways concerning with the hypersensitivity of the individual to foreign substances (15–18). Allergic diseases are a type 2 immune disorder classically characterized by high levels of IgE-mediated inflammation and Th1/Th2 cells imbalance (19–21). The Th 2 immune response involves Th2 cells, type 2 innate lymphoid cells, mast cells, eosinophils, and M2 macrophages (22). Th2 cytokines, particularly IL-4, are essential in the pathophysiology of allergic rhinitis and asthma (23, 24). In type I immediate allergic responses, naïve T cells is activated by dendritic cells to differentiate, proliferate and clonally expand into Th2 cells (23, 25). Enhanced Th2 cytokines induce IgE synthesis in B cells in an indirect manner (26, 27). In turn, IgE can also enhance Th2-cell response after sensitization (28). However, the aberrant immune responses in atopic disorders are not fully understood yet.

Epigenetics plays a major pathogenetic role in the development and management of allergic diseases by superimposing its effects above the DNA molecule through interaction with susceptibility genes, environmental factors, and immunologic influences (29). Epigenetics holds the key to unravel the complex associations between phenotypes and endotypes of allergic disease by identifying effective therapies and diagnosis (30). Epigenetic modifications of genes are contributing to asthma induced by allergens, such as DNA methylation changes in DCs, can be passed to future generations (31, 32). Histone modifications and DNA methylation represent the classical epigenetic mechanisms. Histone modifications participate in airway remodeling by regulation of T cells and macrophages. Inhibitors of histone-modifying enzymes may potentially be used as anti-allergic drugs (33).

## The role of HDAC1 in allergic diseases

HDAC1 displays compensatory or specific roles in different cell types or in response to different stimuli and signaling pathways of atopic disorders. The expression level of HDAC1 in the nasal epithelia is elevated in allergic rhinitis (34), and HDAC1 inhibitors reduce the symptoms of allergic rhinitis (3, 12, 35). Immunohistochemical results also demonstrate the high HDAC1 expression in nasal epithelium of patients with sinusitis and nasal polyps (36). The differentially expressed genes (DEGs) analysis of 1,662 nasal−epithelium tissue samples and 572 DEGs from peripheral blood samples shows that HDAC1 is hub genes and serves an important role in the process of asthma (37). HDAC1 expression is enhanced in patients with severe asthma compared with healthy volunteers (11). Moreover, expression of HDAC1 is upregulated by the stimulation of dermatophagoides pteronyssinus allergen (Der p 1) in peripheral blood mononuclear cells of patients with severe and non-severe asthma (38). Animal models of allergic asthma exhibits significantly higher expression of HDAC1 compared to control. Selective targeting of HDAC1 may improve therapeutic effects of asthma (39). One single nucleotide polymorphism (SNP) in HDAC1 (rs1741981) is closely associated to asthma severity in a recessive model and increases the sensitivity to systemic corticosteroids treatment in asthmatic patients (40, 41). Besides, in epidermal keratinocytes, HDAC1 expression and activity are upregulated by the aryl hydrocarbon receptor nuclear translocator (ARNT or HIF1β) (42).

## Regulation of inflammatory cytokines and downstream protein by HDAC1

**Figure 1:**
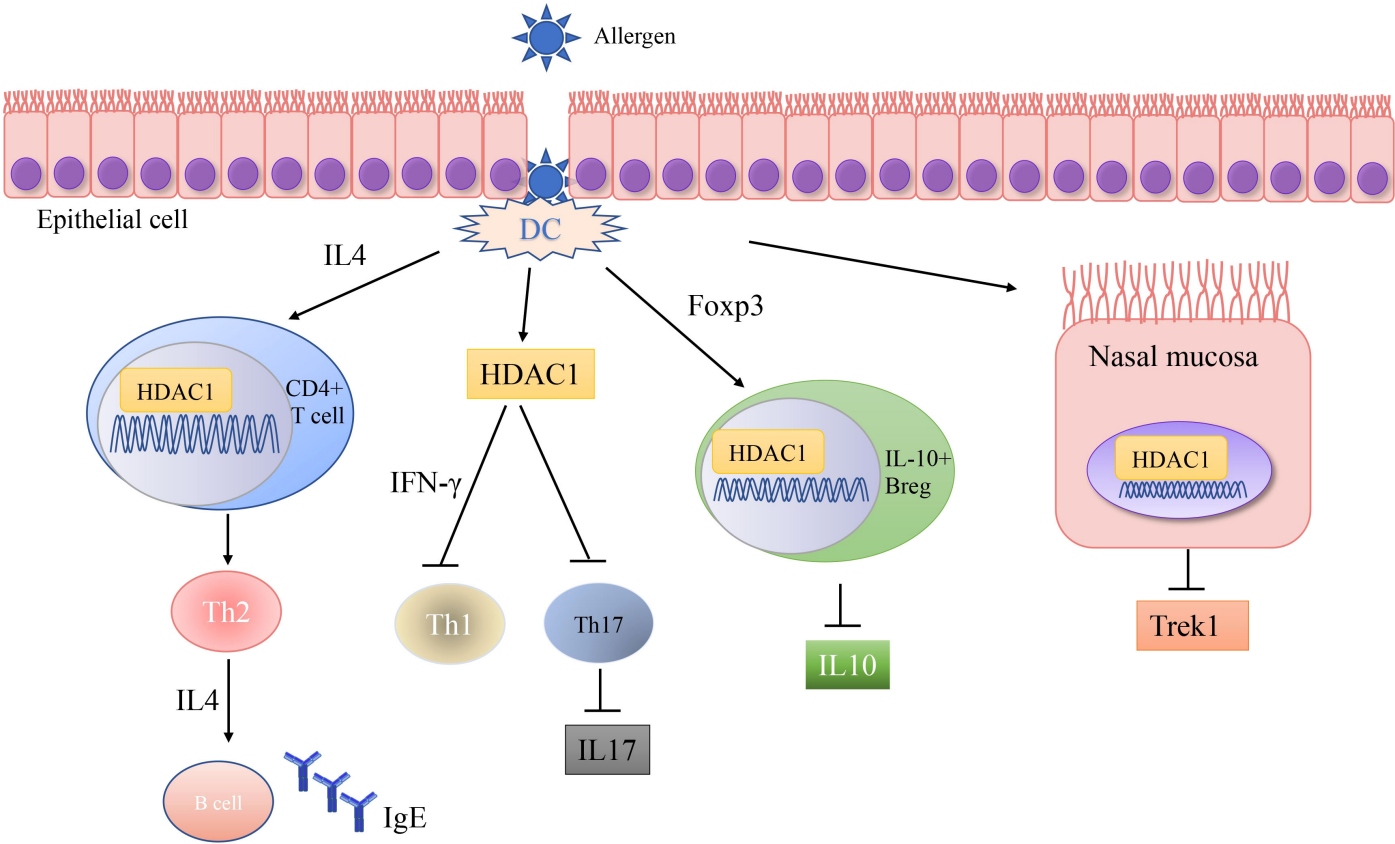
Schematic representations of HDAC1 related mechanism in allergic diseases. Allergic disease patients have an epithelial barrier suffering from allergen stimulation. Exposure to allergens activate dendritic cell and increase HDAC1 expression, leading to significantly increase Th2 cytokine levels, decrease Th1/Th17 cells and anti-inflammatory cytokine IL-10, and Trek-1 expression.

A number of studies have shown that exposure to allergens would increase HDAC1 expression, leading to significantly advanced Th2 cytokine levels, reduced Th1/Th17 cells and anti-inflammatory cytokine IL-10, and Trek-1 expression (**Figure 1**). In the mouse model of allergic rhinitis, epigenetic regulation of HDAC1 produce an imbalance in Th1/Th2 by decreasing the secretion of interferon(IFN)-γ, increasing the secretion of IL-4 and IL-6 (14). Moreover, the transcriptional activity of forkhead box P3(Foxp3) is restrained that decreases T regulatory cells (43). As the number of Th1 cells decreases, the number of Th2 cells correspondingly increases, and subsequently the secretion of IL-4 increases to promote the activation of IgE released by B cells (44). Additionally, murine models of asthma confirm the upregulation of HDAC1 could increase airway inflammation, Th2 cytokine level, IgE and goblet cell metaplasia dramatically (45). Indeed, treatment with HDAC1 inhibitor trichostatin A(TSA) significantly attenuate airway hyper-responsiveness, mucus occlusions in lung tissue and the numbers of eosinophils and lymphocytes in bronchoalveolar lavage fluid. The infiltration of CD4+ and the expression of IL-4, IL-5, and IgE in BALF are also restrained by TSA (13). Particularly, Th2 cytokine interleukin 4 (IL-4) plays a key role in the pathogenesis of allergic disorders (46). HDAC1 can be recruited to the IL-4 gene locus in CD4(+) T cells, thereby promoting the immunoactivity of CD4 positive T cells to increase Th2 cytokine levels (47–49). The IL-4-induced rat nasal epithelial barrier dysfunction is blocked by HDAC1 inhibitor (Trichostatin A), or sodium butyrate (NaB), or administration of Clostridium Butyricum (**Table 1**) (14, 62). A non-secreted IL-4 variant (IL-4δ13) expression in human γδ T-cells is also stimulated by another HDAC inhibitor valproic acid (VPA) (**Table 1**) (58). The Induction of IL-4δ13 increases cytoplasmic IL-4Rα and decreases mature IL-4 (59). Along with the role of HDAC1 in altering the Th2 cytokine profile, it is reported that HDAC1 is recruited to change the euchromatin into tightly-packed heterochromatin to repress its expression in Th17 cells through production of cytokine IL17 (63). HDAC1 inhibitor sodium butyrate increases IL−17、interleukin 2 (IL−2) and interferon γ and decreases the expression of IL−4 and IL−5 (50). HDAC1 regulates the retinoic acid-related orphan receptor-mediated transcriptional activation of IL-17 (64).

**Table 1:** The role of HDAC inhibitor in allergic diseases.

HDACI inhibitor		Structure	Model	Clinical application	Allergic Diseases	References
Trichostatin A (TSA)	pan-inhibitors	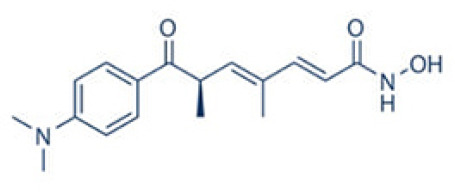	Ovalbumin-induced mouse asthma model;	Phase I clinical trials in hematologic malignancies	Asthma	(13)
Sodium butyrate (SoB, NaB)	selective inhibitors	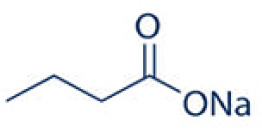	Mouse model of allergic rhinitis	Phase 2 clinical trials in Shigellosis; Randomized controlled trial in inflammatory Bowel Diseases;	Allergic rhinitis	(50–53)
Entinostat	selective inhibitors	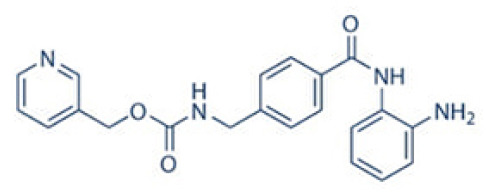	Mouse model of oxazolone-induced contact hypersensitivity	Phase 3 clinical trials in cancer;	Contact hyper sensitivity	(54–57)
Valproic acid (VPA)	selective inhibitors	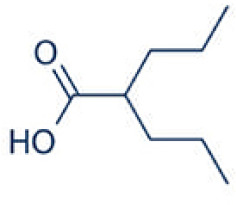	Asthmatic mouse model; Peripheral blood mononuclear cell	Phase 2 clinical trials in cancer	Asthma healthy donors	(58–61)

Apart from the studies showing the Th1/Th2 imbalance and inhibition of IL17, histone deacetylation is an important mechanism that regulates the expression of anti-inflammatory cytokine IL-10 (65). HDAC1 represses IL-10 transcription activity by reducing chromatin accessibility and recruiting histone H3 acetylation at IL-10 regulatory regions (66). Sodium butyrate restrains the activation of HDAC1 in the antigen specific B cells to induce the expression of IL-10 and decrease the production of IgE in allergic rhinitis model (51). Another HDAC inhibitor entinostat stimulates the formation of IL-10 positive Breg cells to suppress contact hypersensitivity *in vivo (* 54). Indeed, the administration with Clostridium butyricum (C. butyricum) enforces the effect of specific immunotherapy on intestinal allergic inflammation by increasing the phosphorylation of HDAC1, the expression of IL-10 and the IgE-producing plasma cells (67).

There are some studies documenting the role of Trek-1 in the maintenance of epithelial cell barrier function (62, 68). The allergic responses induce an insufficiency of Trek1 expression (69). Enhanced IL-4 markedly suppresses the expression of Trek1 *via* upregulating the expression of the HDAC1 in the nasal mucosa of allergic rhinitis (62). The treatment with antigen-specific immunotherapy and administration of probiotic C. butyricum reduce the serum levels of Th2 cytokines by increasing Trek-1 expression levels and decreasing HDAC1 in the nasal mucosa of allergic rhinitis patients (23). Allergic responses markedly suppress the expression of Trek1 in the intestinal epithelia *via* increasing the expression of HDAC1 (70).

## HDAC1 is regulated by exposure to stimuli and is associated with gut microbiome

Different stimuli includes temperature, particles containing hazardous chemicals, and small chemical molecules that exhibits an impact on the expression of HDAC1. Particulate matter (PM) 2.5 exposure and cold stress (PMCS) exposures promote inflammation and redox levels in asthmatic mice through increasing the percentage of Th2 T cells and decreasing Th1 T cells, thereby decreasing HDAC1 expression and hyperacetylation of H3K9 and H3K14 in IL-4 gene promoter of CD4+T cells (71). Mechanically, HDAC1 helps maintain DNA-binding sites (response elements) for redox-sensitive transcription factors by co-repressor complexes (72). Besides, exposure to diesel exhaust particulate matter (DEP) causes degradation of histone deacetylase 1 (HDAC1), thus recruiting histone acetyltransferase (HAT) p300 to the promoter of the Cyclooxygenase-2 (COX-2) gene *in vitro* human bronchial epithelial cell line (BEAS-2B) (73). In addition, chronic exposure to alcohol decreases HDAC1 expression (74). Trichostatin A alleviates tissue damage that is caused by cigarette smoke exposure (75, 76).

On the other hand, HDAC1 is modulated by upstream transcription factors and signaling pathway in allergic diseases. Previous studies have shown that the transcription factor c-Myc-interacting zinc finger protein-1 (Miz1) was upregulated in allergic asthma, which in turn prevented the pro-Th1 skewing through the recruitment of histone deacetylase 1 (HDAC1) and transcriptional repression of IL-12 (77). HDAC1 expression is also increased by the advanced glycation end products *via* the phosphatidylinositol 3-kinase(PI3K)/AKT pathway through promoting the airway inflammation (45).

Moreover, gut microbiome is associated with allergic diseases (78–81). Sodium butyrate treatments lead to increase the richness in the stomach and colon and modify colonic microbial composition in pigs by decreasing HDAC1 (82, 83). The intestinal epithelial cells specific HDAC1 support intestinal homeostasis by controlling specific biological processes including oxidation-reduction, survival and translation processes, differentiation and lipid-related metabolic pathways *via* Janus kinase(JAK)/signal transducer and activator of transcription (STAT) pathway and steroid receptor pathway (84–86).

## Potential of HDAC1 inhibitors as treatments

A large body of evidence shows that HDAC1 is a potential clinical target for treatment of allergic diseases. At present, numerous questions remain regarding to the precise functions of HDAC1 in allergic inflammation. The HDAC inhibitors such as trichostatin A (TSA) have a bidentate cheator, which binds to catalytic Zn2+ (87). The broad-spectrum HDAC1 inhibitor trichostatin A has a hydroxamic acid based structure that affects the expression of thousand genes in the human genome. There is still no clinical application of these HDAC1 inhibitors. Thus, there is an ongoing discussion whether selective HDAC inhibitors have advantage for clinical use. These small-molecule compounds targeting HDAC1 have no serious toxicities.

There are many HDAC inhibitors in ongoing clinical trials (**Table 1**). The study on the tolerance of trichostatin A in patients with recurrent or refractory hematological malignancies is still in progress. Genetic and pharmacological studies have confirmed that HDAC1 is the key enzyme to reverse tumor immune escape. Entinostat selectively promotes the immune editing of new tumor antigens, leading effectively reshaping the tumor immune microenvironment (55). The randomized phase III trial of endocrine therapy confirms target inhibition in entinostat-treated breast cancer patients (56). Valproic acid and entinostat exhibit synergy in preclinical models when combined with rituximab in Non-Hodgkin’s lymphoma (57). On the other hand, Valproic acid is the first-line drug for tonic clonic seizures (60). Besides, Valproic acid induces apoptosis of activated T cells to maintain immune homeostasis, which may be a safe and effective treatment for autoimmune diseases, such as multiple sclerosis (61). Entinostat and valproic acid can potentially be repurposed for treating asthma (88). However, there is no clinical trials to determine the role of entinostat and valproic acid in asthma. These findings highlight the need for further exploration of HDAC inhibitors in allergic diseases.

Sodium butyrate therapy during shigellosis leads to early reduction of inflammation and enhanced antimicrobial peptides (LL-37) expression in the rectal epithelia (52). The double‐blind randomized controlled trial shows that sodium-butyrate supplementation in 49 inflammatory bowel diseases patients increases the growth of bacteria able to produce short‐chain fatty acids (SCFA) with potentially anti-inflammatory action (53). These results support the potential effect of sodium butyrate in modulating gut microbiota, which anyway requires further confirmatory data including more patients. In considering future potential clinical application in allergic diseases, more studies are still needed to develop new HDAC1 specific selective inhibitors. HDAC1 specific selective inhibitors may provide a new starting point for the treatment of allergic diseases.

## Prospective and conclusion

Allergic diseases comprise some of the most common chronic disorders in both childhood and adulthood. Allergic conditions are influenced by epigenetic elements which ultimately affect multiple molecular pathways (89, 90). Accumulating evidences have established in HDAC1 as a critical regulator of immune response in terms of imbalance in Th1/Th2, change in anti-inflammatory cytokine IL-10/IL-17 and Trek-1 expression. Over the past decades, histone deacetylase inhibitors are being evaluated in clinical trials for their safety and efficacy (91, 92). HDAC1 has become an attractive target to treat a wide range of diseases. However, these HDAC inhibitors do not display high selectivity and may restrain related HDACs. The potential side effects due to inhibition of systemic immune response are an urgent problem to be solved. Besides, additional work is required to examine the expression and activity of HDAC1 in allergic diseases. The development of selective HDAC1 inhibitors may lead to new therapeutic agents for allergic diseases, particularly in situations where current therapies are suboptimal.

## Author contributions

All authors contributed to the search and collation of literature. YW contributed to the manuscript preparation and the revision of the manuscript. All authors read and approved the final manuscript.

## Funding

This work is supported by the National Natural Science Foundation of China (NSFC) (Grant 81902331).

## Conflict of interest

The authors declare that the research was conducted in the absence of any commercial or financial relationships that could be construed as a potential conflict of interest.

## Publisher’s note

All claims expressed in this article are solely those of the authors and do not necessarily represent those of their affiliated organizations, or those of the publisher, the editors and the reviewers. Any product that may be evaluated in this article, or claim that may be made by its manufacturer, is not guaranteed or endorsed by the publisher.
